# Phytoplankton diversity explained by connectivity across a mesoscale frontal system in the open ocean

**DOI:** 10.1038/s41598-023-38831-1

**Published:** 2023-07-26

**Authors:** Jørgen Bendtsen, Lykke Laura Sørensen, Niels Daugbjerg, Nina Lundholm, Katherine Richardson

**Affiliations:** 1 Globe Institute, Section for Geobiology, University of Copenhagen, Øster Voldgade 5-7, 1350 Copenhagen K, Denmark; 2 Marine Biological Section, Department of Biology, University of Copenhagen, Universitetsparken 4, 2100 Copenhagen Ø, Denmark; 3 Natural History Museum of Denmark, University of Copenhagen, Øster Farimagsgade 5, 1353 Copenhagen K, Denmark; 4 Globe Institute, Section for Biodiversity, University of Copenhagen, Universitetsparken 15, 2100 Copenhagen Ø, Denmark

**Keywords:** Ecology, Environmental sciences, Ocean sciences

## Abstract

Phytoplankton community composition is important in establishing ecosystem structure and function. Intuitively, we recognize that water movements must be important for modifying spatial gradients and plankton diversity. However, identifying boundaries and exchange between habitats in the open ocean is not straightforward. Here, we use the abundance of nine phytoplankton species closely sampled in a mesoscale frontal system in the northeastern North Sea as a proxy for community composition and explore the relationship between phytoplankton biogeography and transport patterns. Subsurface community distributions could be related to modeled patterns in water movement. A methodology for analyzing pelagic diversity that includes a representation of plankton community composition and an Eulerian connectivity tracer was developed, and the relative importance of connectivity and geographical distance for phytoplankton species composition analyzed. The connectivity tracer identifies timescales and dispersal barriers in the open ocean. Connectivity was found to be superior in explaining pelagic plankton diversity and found to be a prerequisite for understanding the pelagic phytoplankton composition. This approach is a valuable tool for establishing the link between ocean transports, ecosystem structure and biodiversity and for informing the placement of marine protected areas.

## Introduction

With respect to land plants, we intuitively recognize that species composition determines which organisms populate higher trophic levels in local food webs as well as the role of vegetation in local carbon cycling. Much focus is, therefore, directed towards terrestrial plant diversity. In contrast to land plants that all are assigned to a single branch on the tree of life, phytoplankton, which comprise the majority of photosynthesizing life in the ocean, are found in numerous different branches. Thus, phytoplankton, in terms of their genetic origins, differ more from each other than land plants. We, therefore, expect the diversity of phytoplankton communities to have an effect on the heterogeneous distributions of organisms populating higher trophic levels^1^ and in the efficiency of the biologically mediated ocean carbon sequestration^2^. Traditionally, however, it has been difficult to assess and explain phytoplankton diversity distributions at regional and local scales. Access to molecular methods has greatly improved the potential to describe diversity distributions. However, understanding the mechanisms influencing these distribution patterns is still a challenge.

To the human eye, pelagic habitats appear unconstrained by natural boundaries that might be important in determining differences in community distributions. Nevertheless, it is well known that the composition of plankton communities varies spatially from the ocean basin scale^3^ and down to the meso- (~ 10–50 km) and submesoscale^4–7^ (~ 1 km) suggesting that ocean currents modify plankton diversity by acting as transport pathways or dispersal barriers in conjunction with other environmental variables. Global observations of planktonic diversity are more strongly correlated with ocean transit times, i.e., the duration of transport from A to B, estimated from numerical modeling, than other environmental variables^8^. Phytoplankton diversity has also been found to vary across large-scale oceanographic frontal systems^9^ and, accordingly, global model simulations find that correlation length scales among phytoplankton populations are elongated by currents near ocean fronts^10^. Connectivity at the regional scale has also been found to better explain pelagic larval dispersal^11,12^ and genetic differentiation of phytoplankton^13^ than geographical distance. Thus, studies of distributions of different groups of plankton have shown that ocean currents and connectivity are important factors for explaining genetic variation among different sites from global to local scales.

Connectivity between different ocean regions shapes the genetic seascape^14^ and, via the implied interactions between local and immigrating species, it affects ecosystem structure. Furthermore, this influences our capacity for predicting ecosystem services as well as for supporting ocean conservation and management^15,16^, and for mitigating impacts from global warming^17^. In general, connectivity influences pelagic plankton diversity and is important to consider in determining the underlying principles establishing ocean biogeography^18^.

Direct measurements of connectivity in the open ocean require extensive observations of ocean currents and mixing by turbulent eddies. Model simulations, on the other hand, provide a feasible and flexible means of assessing connectivity between different habitats where information on connectivity mainly is limited by model performance. Numerical simulations of connectivity can be based on a “Lagrangian” approach, where particles are released from various locations and information is collected about their arrival times at other locations in the domain^19–21^. Alternatively, connectivity can be evaluated by an “Eulerian” approach, where tracers are included in various subdomains and the subsequent dispersal and presence at other locations express the connectivity in terms of a corresponding relative transit probability^22^. The Lagrangian approach is based on model simulations of water parcels under the influence of ocean currents and mixing, and is suitable for tracking the pathway of individual virtual particles^23^. One advantage of this approach is that behavior, e.g., the life cycle of an organism, can be explicitly associated with each particle, whereas life-cycle behavior must be considered in a statistical sense in Eulerian approaches^24^. However, an Eulerian tracer can be implemented directly in circulation models such that mixing and transport are simulated in conjunction with other model tracers, e.g., salinity. This approach is therefore suitable for describing connectivity of dissolved substances or, similarly, free-drifting plankton. This approach has not previously been applied for simulating connectivity in the open ocean but has been applied in semi-enclosed estuaries^22,25^.

Here, we analyse the relative impact of connectivity and geographical distance on an extensive data set of enumerated plankton species in the northeastern section of the North Sea. Connectivity between pelagic habitats along the shelf-edge frontal system found here is based on an Eulerian method and a methodology is developed to quantify the relationship between diversity and connectivity (“Methods”). From the analysis, we identify dispersal barriers and relevant timescales for phytoplankton communities and discuss the implications of connectivity for biodiversity and genetic diversification of this group of primary producers.

## Results

**Figure 1:**
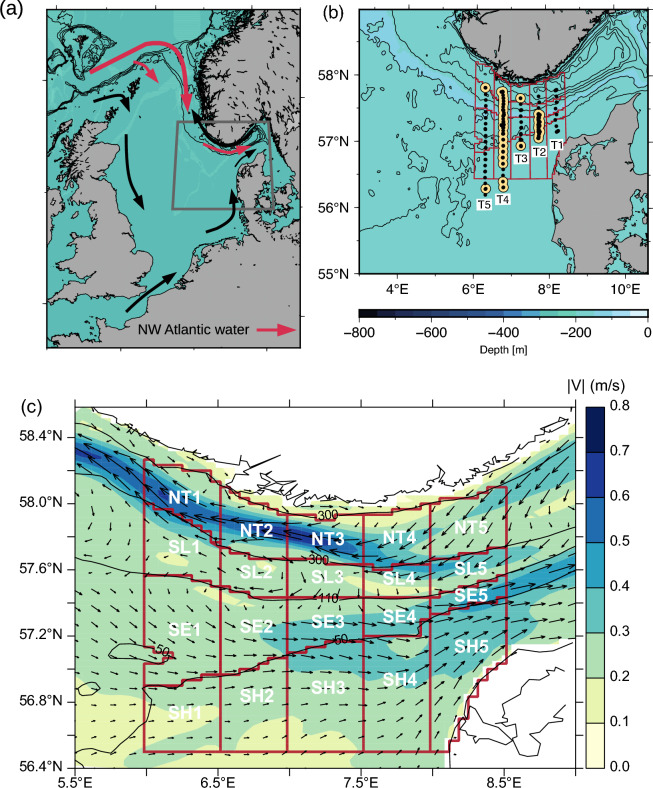
Study area and subdomains. **(a)** Regional circulation showing the origin of subsurface Atlantic water along the shelf edge in the northeastern North Sea^40^. The map was produced by the GMT software^41^. **(b)** VERMIX stations along transects T1–5 with chlorophyll measurements (black bullets) and with phytoplankton species counting (yellow bullets). **(c)** Monthly averaged surface currents simulated in July 2016 (arrows, current speed shown in colors) and bathymetry shown by 50, 110 and 300 m depth contours (black lines). Subdomains for connectivity simulations (red) are referred to as bathymetric sectors along the Norwegian trench (NT1–5), slope (SL1–5), shelf-edge (SE1–5) and above the shallow area (SH1–5).

**Figure 2:**
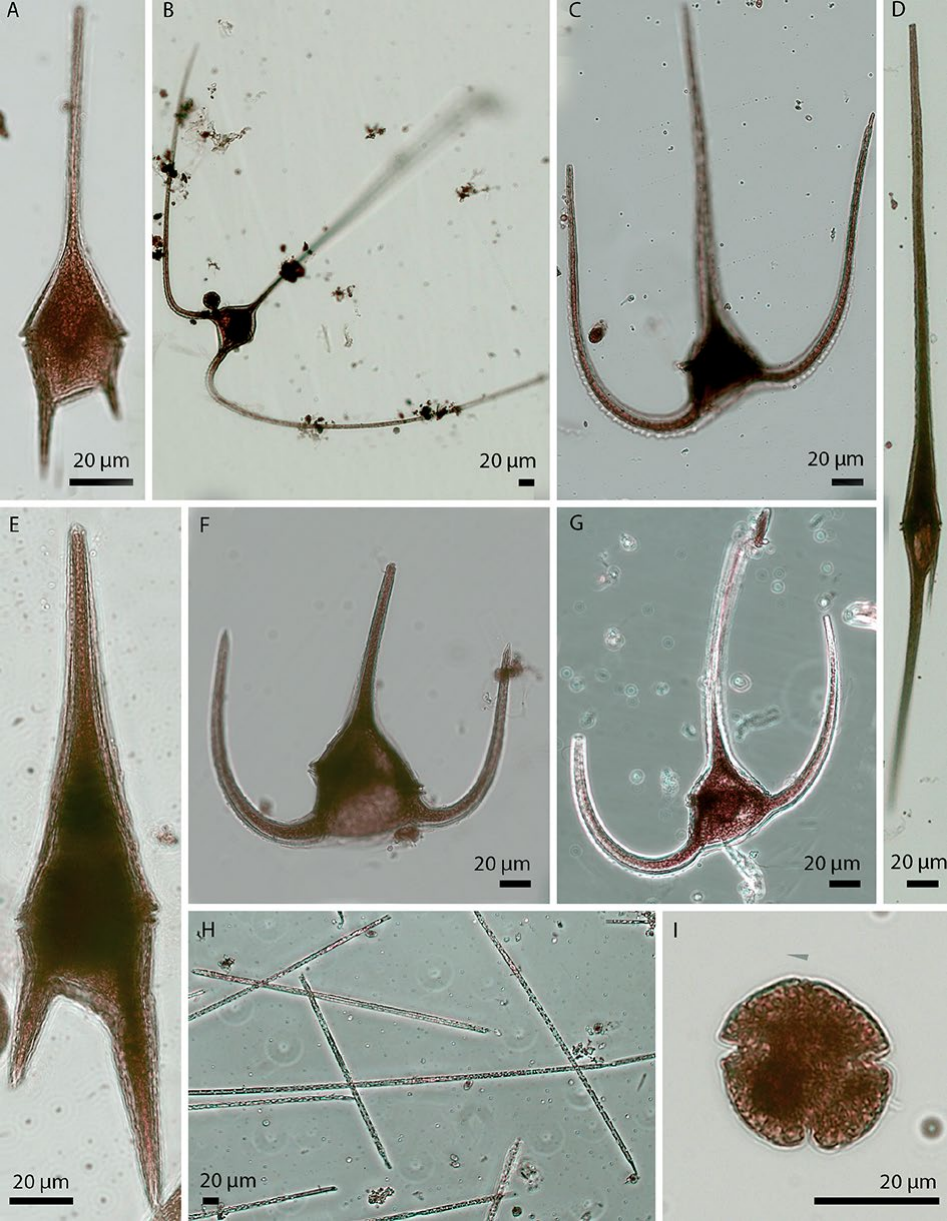
Micrographs of phytoplankters. Light micrographs of Lugol fixed phytoplankters that were enumerated from the samples collected in the North Sea, July, 2016. A. *Ceratium lineatum*. B. *Ceratium macroceros*. C. *Ceratium horridum*. D. *Ceratium fusus*. E. *Ceratium furca*. F. *Ceratium tripos*. G. *Ceratium longipes*. H. *Proboscia alata*. I. *Karenia mikimotoi*.

**Figure 3:**
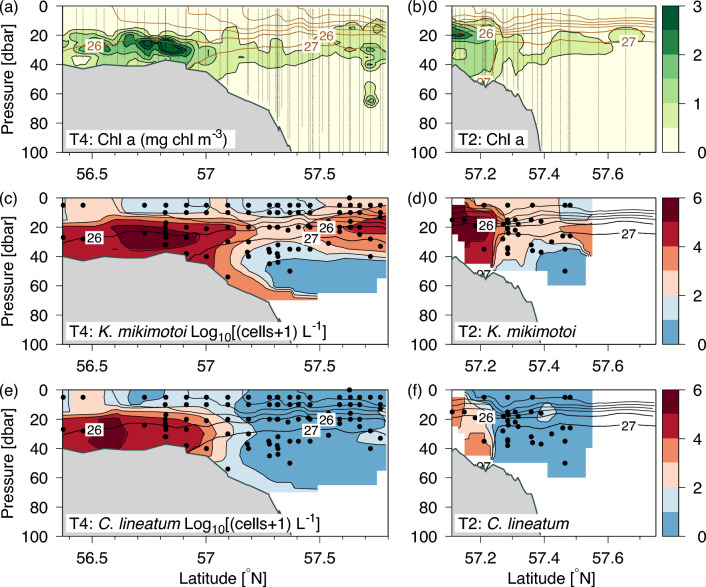
Distributions of dominating species. Distributions along transect 4 (T4: 6.75° E) and 2 (T2: 7.75° E): **(a,b)** Chlorophyll *a*, based on chlorophyll *a* calibrated fluorescence measurements, and density contours (brown lines, σ_θ_, intervals of 1 kg m^−3^), **(c,d)** concentration of *Karenia mikimotoi* and (**e,f**) *Ceratium lineatum*. Dots show locations of the phytoplankton species enumerations.

Model simulations of the mean surface current showed an eastward transport along the shelf edge and an outflow from the Baltic Sea with increased westward current speeds above the central part of the Norwegian Trench (Fig. 1).The shelf-edge frontal system separated sub-surface water of Atlantic origin above the Norwegian trench from the less dense North Sea water above the southern shallow area^26^_._ Abundances of 9 large and easily identifiable phytoplankton species (yet one, *K mikimotoi*, confirmed by DNA sequencing) were used to identify phytoplankton communities (Fig. 2). Chlorophyll *a* distributions showed a characteristic pattern with low concentrations in the surface layer, relatively high subsurface concentrations above the shallow shelf (> 2 mg chl *a* m^−3^) and a subsurface chlorophyll *a* maximum (around the 27 kg m^−3^ isopycnal) extending from the shelf-edge zone towards the deeper areas (Fig. 3a, b).

### Phytoplankton species distribution

Cell concentrations were analyzed and enumerated (Fig. 3, Fig. S1–S3). *Proboscia alata* was present in 98% of the samples with an average and maximum concentration of 1600 and 32,600 cells L^−1^, respectively. *Karenia mikimotoi* and *C. lineatum* were the two most abundant species and they were represented in 91% and 42% of the samples, respectively. Their average concentrations were above 10,000 cells L^−1^ and the maximum values found for the two species were 740,000 and 330,000 cells L^−1^, respectively. In total, nine species were present in more than 30% of the samples. Two species (*C. pentagonum* and *C. buchephalum*) were only present in 4% of the samples.

The two most abundant species showed a characteristic distribution across the shelf edge: *C. lineatum* was primarily found above the shallow shelf whereas *K. mikimotoi* was found not only above the shallow shelf areas but also above the deeper areas (Fig. 3c–f). The ubiquitous *P. alata* had its largest concentration above the deeper areas. *Ceratium fusus* was found along most of the transects in approximately equal amounts and *C. tripos* was mainly present above the shelf edge and deeper areas (Fig. S1). *Ceratium furca* and *C. horridum* showed a bimodal distribution with a minimum above the shelf edge whereas *C. macroceros* was found along most of the western transect (T4). This latter species, however, also showed a bimodal distribution with a shelf edge minimum on transect 2 (Figure S2). *Ceratium longipes* was mainly found above the deeper areas (> 50 m) and the two least abundant species, *C. pentagonum* and *C. buchephalum,* were found at a few stations in the shelf edge zone (Fig. S3). Thus, the nine species represented diverse distributions in the area with some indications of separation between the shallow shelf and the deeper areas and possibly a unique shelf edge community (Table S1).

### Connectivity in the northeastern North Sea

**Figure 4:**
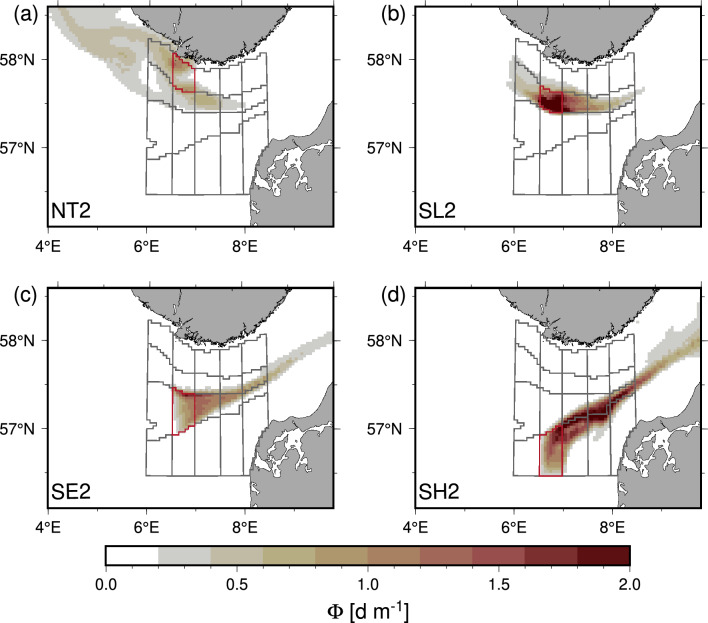
Simulated tracer connectivity. Model simulation 31st of July of the vertically integrated tracer connectivity (Φ [d m^−1^], see “Methods” Eq. 1) in the upper 50 m and initialized the 1st of July in **(a)** subdomain NT2, **(b)** SL2 **(c)** SE2 and **(d)** SH2 (initial domains indicated with red color).

The connectivity tracer (Φ, “Methods”) quantifies the connectivity from a location to the domain where it originated (domains shown in Fig. 1c). For example, in the stationary case where the connectivity tracer remains in the domain where it was initialized (i.e., φ(t) = 1 m^−3^, “Methods”), the vertically and temporally integrated tracer would have a value of ~ 1550 d m^−2^ after 1 month, (i.e., the product of the initial concentration, the depth of the surface layer of 50 m and the time interval of 31 days). Thus, the integrated tracer is closely related to dilution by mixing and transport by the mean current field: areas with a large exchange result in low concentrations. By the end of July, the integrated tracers were generally below 3 d m^−2^, corresponding to ~ 0.2% of the initial concentration. Examples originating in four different domains are shown in Fig. 4. High dispersal close to the Norwegian coast resulted in a low concentration of Φ (e.g., in NT2, Fig. 4a), whereas the less dynamic area above the slope (e.g., SL2, Fig. 4b) was characterized by a relatively low dispersal. Material above the shelf-edge was seen to be transported rapidly eastward (Fig. 4c) whereas a smaller dispersal characterized the eastward flow above the shallow area (Fig. 4d). A clear separation is indicated between the shelf edge and the more shallow North Sea area.

A characteristic time scale for the exchange of matter within each domain was obtained from the exposure time in the initial volume (i.e., i = j in Eq. 2). The exposure time in the 20 domains ranged between 0.3 days (SE5) and 3.3 days (SL3) with an average of 1.3 ± 0.3 days and showed that a large exchange with neighboring areas characterized the entire study area. For example, an exposure time of 1.1 days implies that practically all (> 99%) water within domains along the shelf edge is replaced within a week.

### Diversity across the shelf edge

We tested the hypothesis that ocean currents along the four bathymetric sectors (i.e., east–west directed rows of domains: SH1-5, SE1-5, SL1-5 and NT1-5, “Methods”) tend to reduce differences between plankton communities. The potential relation between diversity and connectivity was investigated from an NMDS-analysis of the dissimilarity matrix of the plankton samples. The distribution in NMDS-space was analyzed with respect to the four bathymetric groups and the ANOSIM statistics showed that the most significant grouping was obtained when only data below 20 m depth were included (R = 0.53, p = 10^–4^). Including data deeper than 10 m resulted in a less significant grouping (R = 0.30) where the lower R-score indicates a larger overlap between the four bathymetric sectors. Including data from the surface resulted in a non-significant grouping (R = 0.18). Possible explanations for this finding are that surface waters were nutrient-depleted and phytoplankton abundance generally very low. Furthermore, size-fractionated chlorophyll *a* determinations (data not shown) indicated that there were few of the large cells we use here to characterize phytoplankton communities found in this layer. We used, therefore, only data from below 20 m for analyzing the relationship between plankton dissimilarity and connectivity (n = 58).

**Figure 5:**
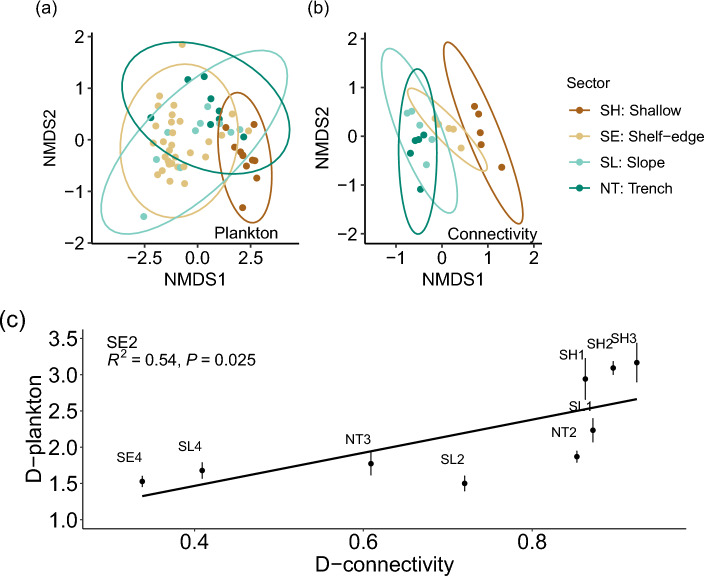
Phytoplankton diversity and dissimilarity index. Two-dimensional NMDS plots based on the Bray–Curtis dissimilarity index of **(a)** phytoplankton samples deeper than 20 m (stress = 0.097, n = 58) and **(b)** connectivity tracers between the 20 domains (stress = 0.096). Colors represent location of samples **(a)** or domains **(b)** in the four bathymetric sectors, and ellipses enclose 95% confidence levels assuming a bivariate normal distribution. **(c)** Average dissimilarity distances in ecological space (bullets and standard errors with linear regression) versus connectivity distance (see “Methods”) between the different domains and SE2.

The corresponding NMDS-plot of dissimilarity (Fig. 5a, stress = 0.097) showed that samples above the shallow shelf were separated from samples above the shelf-edge, although some overlap is indicated by a few samples from the shelf-edge being located in the shallow regime. Samples from the slope and trench-area are, in general, grouped separately in the NMDS-space and the overlap for these few samples potentially indicates water exchange between these two sectors.

### Connectivity in the northeastern North Sea

NMDS-analysis of the connectivity matrix resulted similarly in a significant separation between the four bathymetric groups (ANOSIM R = 0.64, p = 10^–4^) with some overlap between the slope (SL1–5) and trench (NT1–5) sectors, a more isolated shelf (SH1–5) sector and some connections between the shallow and deep area via the shelf-edge (SE1–5) sector (Fig. 5b, stress = 0.096).

The location of domains within each group in the NMDS-space was also consistent with their relative distribution in the area. For example, the westernmost domain in the shallow sector (i.e., SH1, the point with the largest NMDS2 coordinate in Fig. 5b) is located furthest away from the easternmost domain in that sector (i.e., SH5, largest NMDS1 value), and the other shallow domains are located sequentially in between. The shelf-edge domains are similarly located sequentially from SE1 and eastward (i.e., from the top towards the bottom in the figure). Domains along the trench-sector (NT) show that the easternmost domain (i.e., NT1, smallest NMDS2-value) is relatively isolated. This indicates increased exchange between sectors NT2–NT5. The slope domains are, to some extent, mixed with domains from the trench-sector and the westernmost domain (SL1, i.e., lowest NMDS2 value within the SL-group) is again located farthest away from the more closely spaced SL2–5. Thus, the two-dimensional NMDS analysis of the connectivity matrix provides a simple representation of connectivity between the various domains in accordance with their relative position and the mean circulation in the area.

### Explaining similarity with connectivity and distance

Connectivity provided a better measure (R^2^ = 0.54, p = 0.03) for understanding spatial variation of phytoplankton communities than physical distance (R^2^ = 0.05, p = 0.56). Statistical tests of these relationships were made for all domains with plankton samples. In total, 58 plankton samples from below 20 m in nine domains were analyzed and the number of samples within each domain varied between 1 and 18 (Table 1).

**Table 1:** Correlations with connectivity.

Domain	N	Connectivity R^2^	Physical R^2^
NT2	6	0.67**	0.61*
NT3	1	0.13	0.06
SL1	2	0.07	0.01
SL2	3	0.72**	0.52*
SL4	5	0.76**	0.49*
SE2	18	0.54*	0.05
SE4	12	0.05	0.30
SH1	1	0.06	0.13
SH2	10	0.14	0.05

Linear regression of plankton diversity based on distances in the two-dimensional NMDS-space between all samples (N) in a domain compared with all samples from the other domains, versus the corresponding distances in connectivity-space or physical space. R-squared of linear regressions are calculated for both connectivity and physical distances. Significance levels of p < 0.05 and p < 0.01 are shown with superscript * and **, respectively. All domains with plankton samples below 20 m are included.

Four domains showed a significant linear regression between dissimilarity and connectivity (i.e., NT2, SL2, SL4 and SE2) and connectivity explained more than 50% of the variance. In three of these four domains, there was also a significant relationship between dissimilarity and physical distance (i.e., NT2, SL2 and SL4). In general, there was a better explanation of the total variance by connectivity (R^2^ ranging between 0.54 and 0.76) than physical distance (R^2^ between 0.49 and 0.61). For those stations where community dissimilarity could be significantly explained by physical distance, significance was only achieved at the 0.05 level whereas the significance levels to connectivity were below 0.01.

Three subdomains only contained one or two samples (NT2, NT3, SH1) and showed no significant correlation to connectivity or geographical distance, possibly due to a poor representation of the plankton community in the entire subdomain by the few samples. Subdomain SH2 (10 samples) did not show correlation to neither connectivity or distance, and this could be explained from the general eastward transport in SH2 and the location near the western edge of the subdomains. Thus, this domain experienced a limited influence from the other domains due to the eastward transport, in accordance with the low significance of the correlations. Subdomain SE4 (12 samples) similarly showed no significant correlation to connectivity or distance, however the closest subdomain in connectivity space was SE2 (not shown) in accordance with the general transport along the shelf edge (Fig. 1). This indicated that samples from this central domain was more exposed to mixing from several sectors (Fig. 4).

Analysis of diversity between individual domains also showed that connectivity, in general, provided a better measure for understanding spatial variation than physical distance. For example, the average dissimilarity of plankton samples in the western part of the shelf-edge domain (SE2, Fig. 5c) was relatively distant from the nearby shallow domains (SH1, SH2 and SH3). This was in good accordance with the low connectivity to these areas. Similarly, the close connection between the shelf-edge domains SE2 and SE4 was in good accordance with the corresponding small distance in plankton dissimilarity space. Another example was made for the SL4 domain (Fig. S4) located in the eastern part of the slope sector where dissimilarity was closely related to connectivity (R^2^ = 0.76, p < 0.005) whereas a weaker relationship was found to physical distance (R^2^ = 0.49, p = 0.035). In summary, linear regression showed that connectivity was a better explanatory factor than physical distance and it also provided a more relevant measure for understanding small-scale variation of diversity in the area.

## Discussion

This study shows connectivity, determined here by an Eulerian method, to be a superior explanatory factor than physical distance in explaining the distributions of phytoplankton communities in the North Sea. Connectivity in marine waters has previously been determined by Eulerian methods for semi-enclosed estuarine areas but, as far as we know, this is the first example where the approach is used in more open marine waters. Understanding and mapping phytoplankton diversity are potentially important, e.g., for determining the ecological role of these organisms. However, phytoplankton are not the only organisms whose distributions are potentially influenced by water movements. Many bottom-living species have, for example, pelagic larvae. We, therefore, argue that the Eulerian approach to describe connectivity applied provides a potentially valuable tool in selecting locations for the placement of protected areas designed to protect marine biodiversity.

### Connectivity between pelagic habitats

The fact that pelagic habitats are unconstrained by physical boundaries constitutes a challenge when analyzing interactions between dynamics and biological growth and decay in plankton communities. In the case of pelagic communities, however, we argue that an Eulerian approach provides advantages and is complementary to corresponding Lagrangian methods because connections between entire volumes are considered. The connectivity tracer provides a direct measure of connectivity between different domains in the open ocean. In addition, this method provides relevant timescales for growth and decay of phytoplankton between domains in terms of exposure time. The distribution of the connectivity tracer thus represents an integrated measure of mixing and transports between separated volume entities. Furthermore, the study shows a low-order projection of the connectivity matrix was in accordance with the general circulation in the area. Thus, connectivity was superior in explaining plankton diversity and connectivity is therefore a prerequisite for understanding the distributions of phytoplankton in this area. As there is no reason to believe the methods employed here would not be applicable to other more open marine regions, we advocate for more generally including connectivity in the analysis of plankton diversity and pelagic ecosystem structure.

### Connectivity and genetic diversity

Connectivity impacts the composition of phytoplankton communities and, in addition, may also influence genetic diversity of individual species^27–29^. Similarly, gradients in genetic variability may be established in the open northeastern North Sea where reduced connectivity keeps the shallow shelf and deeper parts above the Norwegian Trench separated across the shelf edge. Connections between these relatively closely spaced habitats would mainly be via the general large-scale cyclonic circulation in the area with a timescale of weeks or months. Thus, the shelf edge front acts as a barrier by separating species and populations for many generations and potentially for the entire growth season. Such a lack of connectivity will support genetic diversification. We therefore speculate that, in addition to the relationship demonstrated here between connectivity and phytoplankton community composition, there will also be significant species- and population-specific genetic differences across the shelf edge, e.g., differences among populations of *K. mikimotoi*, *C. furca* and *C. horridum* found above the shallow shelf and the trench.

### Connectivity as a potential management tool

Following the 2022 Conference of the Parties (COP) of the UN Convention of Biological diversity, where the goal of placing 30% of marine (and terrestrial) area under some form of protection, an increased interest in establishing Marine Protected Areas (MPAs) can be expected. There is, however, little empirical evidence as to how these areas can best be located in order to support biodiversity and ecological processes. On a global scale, connectivity (applying a Lagrangian approach) has been used to identify coral spawning sites critical to protect in efforts to maximize the resilience of coral reefs to climate warming^30^. In that case, the location and behavior of the particles (coral spawn) introduced to the water column is known and bounded in space. Similarly, we argue that the Eulerian approach used here can be of value in selecting sites for future MPAs by considering the connectivity between pelagic habitats in the open ocean. The advantage with this approach is that it can provide a generic understanding of the potential distribution of pelagic organisms in a given region. Thus, it can potentially be applied to ensure that highly diverse regions and the locations from whence the organisms they recruit originate are protected.

## Conclusion

Dispersal barriers for pelagic marine organisms exist in the form of fronts and ocean currents. In the absence of such barriers, connectivity between two ocean sites will be large and vice versa. When connectivity is low, phytoplankton communities can be expected to develop relatively independently from one another. Here, a new method is outlined and implemented for quantifying the impact of connectivity on plankton diversity in open ocean regions based on an Eulerian connectivity tracer. The approach applied on phytoplankton species in the northeastern North Sea identified the shelf-edge as a dispersal barrier and showed connectivity to be a better explanatory factor for phytoplankton biodiversity in this area than physical distance. This Eulerian approach calculates connectivity and relevant timescales for plankton communities between entire sub-volumes in the open ocean and is therefore complementary to traditional Lagrangian particle-tracking methods. Connectivity was found to be essential for understanding links between ocean transport and plankton distributions and thereby ecosystem biodiversity. Thus, this approach can also potentially prove to be a valuable tool in the identification of sites relevant for the establishment of Marine Protected Areas.

## Methods

### Observations from VERMIX

The VERMIX study was carried out on board R/V Dana during the period 12–31 July 2016 and along five transects (T1:5) in the northeastern North Sea (Fig. 1). The study area covered the relatively deep (~ 500 m) Norwegian Trench, the shelf edge zone and parts of the more shallow North Sea. In total, 132 stations were sampled with measurements of hydrography and chlorophyll *a*^26^.The depth of the euphotic zone was defined as 50 m (determined from the average 0.1% PAR level from all stations in the area). A previous analysis of chlorophyll a distributions along the five transects showed a consistent pattern at all transects with high concentrations above the shallow shelf area and a subsurface chlorophyll a maximum above the slope area (Fig. 3a)^26^.

### Plankton samples

Phytoplankton samples were collected from depths ranging between 5 and 50 m at 47 different stations (127 samples) mainly along two transects, T2 and T4 (Fig. 1b). Prior to microscopic enumeration the samples were filtered through a 500 µm filter to remove larger zooplankton and fixed in acidic Lugol’s iodine (final solution 3%) before being stored in darkness at room temperature during the cruise and afterwards at 4 °C for 2–5 months. Cell counts were performed on seven species of *Ceratium* (i.e., *C. lineatum*, *C. fusus*, *C. macroceros*, *C. tripos*, *C. furca*, *C. horridum* and *C. longipes*), *Karenia mikimotoi* (dinoflagellates) and *Proboscia alata* (centric diatom). Identification of *Karenia mikimotoi* was confirmed by ITS rDNA sequence determination whereas the other species were identified morphologically. Using 50 mL sedimentation chambers, species were enumerated using the Utermöhl method^31,32^ and based on counting at least 400 cells of each species, or all the cells when < 400 were present in the samples. All cells were counted at 100× magnification. Micrographs of all nine species are shown in Fig. 2.

### Model description

An ocean model, based on the three-dimensional primitive-equation COHERENS circulation model^33^, was applied for calculating connectivity between stations in the study area. The model covered the entire North Sea and Baltic Sea with a horizontal and vertical resolution of ~ 3.7 × 3.7 km and 20 stretched vertical layers, respectively^34^. The model was driven by meteorological forcing and transports through the open boundaries, and the model simulation in July 2016 was started from an initial 6 month spin up period^34^.

### Connectivity tracer

Connectivity (Φ_i,j_) between different areas is calculated from the distribution of passive (i.e., no internal sinks and sources) connectivity tracers (φ_i_) initialized (i.e., at time t = 0) in different subdomains (Ω_n_, n = 1:N) in the study area (Fig. 1). Tracers have an initial value of one in their initial domain (φ_i_(t = 0, Ω_n=i_) = 1) and zero elsewhere (φ_i_(t = 0, Ω_n≠i_) = 0) and are integrated with a baroclinic time step (50 s, i.e., similar to the time step applied for temperature and salinity).

In general, a spatial and temporal integral of the tracer φ_i_ within a domain Ω_j_ can be determined from:

1 $${\Phi }_{i,j}=\int\limits_{{\Omega }_{j}} \int\limits _{0}^{\infty }{\varphi }_{i}dtd\Omega$$

A subdomain exposure time (T_i,j_) can then be defined by normalizing with the initial amount of the tracer^22,23^:

2 $${T}_{i,j}=\frac{{\Phi }_{i,j}}{{\int }_{{\Omega }_{i}}\varphi \left(t=0\right)d\Omega }$$

The exposure time, T_i,j_, expresses the duration that a tracer originating from a subdomain (Ω_i_) is within another subdomain (Ω_j_). For example, in the case where the initial subdomain itself is considered, i.e. *j* = *i*, then T_i,i_ ranges between zero and infinity, corresponding to the cases where all the material disperses immediately or some of the material remains in the subdomain, respectively. Exposure time is related to the residence time that expresses the average time material is within a domain^35,36^ and, in the case where *j* = *i*, the two time scales become identical in the limit where no water re-enters the domain after passing through it. In general, the exposure time represents the amount of time a tracer is within a domain including periods where a tracer re-enters the domain, and exposure time is therefore always larger than or equal to the residence time. When the two domains are different (i.e., j ≠ i in Eq. 2), the exposure time expresses the time spent by material from subdomain Ω_i_ in subdomain Ω_j_ and, in principle, it will be between zero and infinity in the extreme situations where none of the material from Ω_i_ passes through Ω_j_ or where some material goes into and remains in Ω_j_, respectively. The exposure time is therefore a relevant time scale to consider for analyzing the impact from immigrating phytoplankton species on the local plankton community composition, when the impact can be assumed to be proportional to the time spent by the plankton species in the new domain. This assumption implies that impact from immigrating phytoplankton is both direct via their presence and indirect via their growth and cell division. Integrals of the connectivity tracer are integrated vertically from the bottom of the euphotic zone to the surface at every timestep in the entire model domain. The connectivity (Φ_i,j_) is subsequently calculated for the various domains.

### Initializing connectivity tracers

Connectivity tracers are initialized in the euphotic zone in 20 subdomains covering the study area between 56.5° N and 57.8° N and 6.0° E–8.5° E (Fig. 1c). Each subdomain is a half degree longitude wide, centered around the five transects (6.25° E, 6.75° E, 7.25° E, 7.75° E and 8.25° E) and defined in four bottom depth intervals: < 50 m, 50–110 m, 110–300 m and > 300 m. The domains covered all stations except for the three southernmost stations that were located just outside the southern boundary of their domains. The subdomains (from north to south) are referred to as: (NT1–5) located above the Norwegian trench, (SL1–5) located above the slope region, (SE1–5) along the shelf edge zone and (SH1–5) above the shallow area located south of the shelf edge. Each east–west directed row of domains (e.g., SL1–5) is referred to as representing different bathymetric sectors. The selection of subdomains were motivated by the simulated along-shelf circulation pattern^34^ (Fig. 1c) and the distribution of watermasses^26^.

The stretched vertical model grid implies that the bottom of the model layer in the euphotic zone varies between 40 and 48 m depth in the various domains, except for domains above the shallow area (i.e., SH1–5) where the euphotic zone is deeper than the bottom depth (i.e., between 28 and 43 m in SH1–5). Variations in bottom depth between the different domains due to the vertical grid spacing are not critical for the analysis because differences in domain volumes are accounted for in calculating the exposure time (cf. the denominator in Eq. 2).

### Connectivity matrix for plankton

Connectivity can be expressed by the exchange of material (p_i,j_) from one location (i) to another (j), and, from this, a connectivity matrix (P_i,j_) can be formed by normalizing with the sum of exchange from a specific location (i) to all other localities in the domain under consideration, i.e., P_i,j_ = p_i,j_/Σ_i_p_i,j_. Thus, the elements in the connectivity matrix express the relative probability that a connection is established.

Here, we apply the exposure time as representing the exchange by assuming that the probability of an impact from introduced plankton on the plankton community composition at another location is proportional to the time plankton are present in the area, i.e., the exposure time. We normalize the exposure time by a time scale (τ) for plankton dispersal. The subsequent analysis is not sensitive to this normalization, however, it provides a non-dimensional probability. Our interest is to determine the importance of dispersal during the stratified growth season for plankton diversity and, therefore, we chose a time scale of one month and approximate the exposure time during this finite period in Eq. (1). This limitation will, in most cases, only have a minor influence on Φ when the neighboring domains are within a distance traveled by water parcels in less than a month. Finally, the probability is calculated per area to ensure that probabilities can be compared between neighboring domains of different sizes. Thus, a probability per surface area (A_j_) that material from domain Ω_i_ is present in domain Ω_j_ during the time period (τ) can then be estimated from:

3 $${P}_{i,j}=\frac{{T}_{i,j}}{{A}_{j}\tau }$$

The elements (P_i,j_, in units of m^–2^) constitute the connectivity matrix. This expression can be compared with connectivity derived from Lagrangian model simulations (e.g., the release of particles) where particles observed at a location (j) are divided by the total number of particles dispersed from another location (i). The total number of released particles corresponds to the initial distribution of a connectivity tracer, cf. the division by the volume integral of the initial tracer distribution in Eq. (2).

### Similarity and connectivity distances

Similarity between plankton samples was analyzed from a distance matrix based on the Bray–Curtis dissimilarity index (BC_i,j_) of the total number of enumerated species; BC_i,j_ = Σ_n_|s_n,i_ – s_n,j_|/Σ_n_(s_n,i_ + s_n,j_), describing the dissimilarity between two samples (i,j) where summation over the abundance (s_n,i_) of all the species (n = 9). BC_i,j_ is an index between zero and one, corresponding to the two extremes of identical or completely different species compositions, respectively. The distance matrix was not standardized before the transformation and two-dimensional scaling (i.e., an NMDS-analysis with only two axes) was found to be significant and therefore preferred for simplifying further analysis.

Similarly, a two-dimensional NMDS-analysis of the connectivity matrix (Eq. 3) resulted in a significant representation of the connectivity-probability between the various domains. Connectivity was approximated from this NMDS representation by associating each sample with their respective domains and calculating the corresponding distance between these two domains in the NMDS-space.

Dissimilarity, in terms of distance between samples in the NMDS-analysis, was compared with both connectivity and physical distance. Statistics of linear regressions of dissimilarity distance versus either the corresponding connectivity or physical distance were evaluated for quantifying their relative impact on phytoplankton diversity.

The significance of both connectivity and physical distance with respect to the observed phytoplankton diversity was tested. Plankton samples within a domain were compared with samples from all other domains with plankton samples and the average diversity between the samples was allocated to the respective domain pairs. For example, all samples from SE2 were compared to all samples from SE4 and the dissimilarity-distances in the two-dimensional NMDS-space were calculated (i.e., the distances between the corresponding data-points belonging to SE2 and SE4 in Fig. 5a). The distance between all these sample pairs were averaged and this provided an average dissimilarity between the two domains. A corresponding distance in connectivity-space was calculated in a similar manner from the NMDS-ordination of the connectivity matrix (Fig. 5b). Finally, the physical distance between the sample pairs from the two domains was calculated from the geographical position of the two stations and the average of all samples within the domains characterized the physical distance between samples from the two domains. Similar relations were made for SE2 and all other domains with plankton samples, e.g., SE2–NT2, SE2–NT3, etc (Table 1). The statistical relationships were then tested by linear regression between dissimilarity versus connectivity (Fig. 5c) and physical distance (Fig. S4).

The NMDS-analysis and ANOSIM were made in R^37^ using the VEGAN-package^38^ and statistical tests based on Welch modified two-sample t-test were based on the BSDA-package for statistics^39^.

## Supplementary Information

Supplementary Information.

## Data Availability

Data with enumerated phytoplankton species are in Supplementary Information.
