# Supplementary material for: Phytoplankton diversity explained by connectivity across a mesoscale frontal system in the open ocean
Source: Sci Rep. 2023 Jul 26;13:12117. doi: 10.1038/s41598-023-38831-1 (PMC10371993; doi:10.1038/s41598-023-38831-1)
Supplement: Supplementary file 1 — Supplementary Information. [file 41598_2023_38831_MOESM1_ESM.pdf]

Supplementary Information

Phytoplankton diversity explained by connectivity across a mesoscale frontal system in the open ocean

Jørgen Bendtsen[1,*], Lykke Laura Sørensen[2], Niels Daugbjerg[2], Nina Lundholm[3] & Katherine Richardson[4]

[1]Globe Institute, Section for Geobiology, University of Copenhagen, Øster Voldgade 5-7, DK-1350 Copenhagen K, Denmark

[2]Marine Biological Section, Department of Biology, University of Copenhagen, Universitetsparken 4, DK-2100 Copenhagen Ø, Denmark

[3]Natural History Museum of Denmark, University of Copenhagen, Øster Farimagsgade 5, DK-1353 Copenhagen K, Denmark

[4]Globe Institute, Section for Biodiversity, University of Copenhagen, Universitetsparken 15, DK-2100 Copenhagen Ø, Denmark

*Corresponding author at: Section for Geobiology, University of Copenhagen, Øster Voldgade 5-7, DK-1350 Copenhagen K, Denmark, Tel: +45 2167 1535

E-mail address: jorgen.bendtsen@sund.ku.dk

Table legends

Table S1 Enumerated phytoplankton. Large and easily identifiable dinoflagellates (8 species) and a single diatom species were enumerated in an inverted microscope using the Uthermohl method. The sedimentation volume was 50 mL. When possible, at least 400 individuals of each species were counted. Otherwise, the total number of individual species present in the plate chamber was used.

Figure Legends

Figure S1 Phytoplankton distributions. Distributions along transect 4 (T4: 6.75 ºE) and 2 (T2: 7.75 ºE): (a, b) concentration of *Ceratium fusus* and density contours (brown lines, σ_θ, intervals of 1 kg m^{-3}), (c, d) concentration of *Ceratium tripos* and (e, f) *Proboscia alata*.

Figure S2 Phytoplankton distributions. Distributions along transect 4 (T4: 6.75 ºE) and 2 (T2: 7.75 ºE): (a, b) concentration of *Ceratium macroceros* and density contours (brown lines, σ_θ, intervals of 1 kg m^{-3}), (c, d) concentration of *Ceratium furca* and (e, f) *Ceratium horridum*.

Figure S3 Phytoplankton distributions. Distributions along transect 4 (T4: 6.75 ºE) and 2 (T2: 7.75 ºE): (a, b) concentration of *Ceratium longipes* and density contours (brown lines, σ_θ, intervals of 1 kg m^{-3}), (c, d) concentration of *Ceratium pentagonum* and (e, f) *Ceratium buchephalum*.

Figure S4 Connectivity and Euclidean distances. Average dissimilarity distances in ecological space (bullets and standard errors) versus connectivity distance between samples in (a) SE2 (also shown in Figure 4c), (b) SL4 and all other samples, and (c, d) the corresponding relation to Euclidean distances (linear regression, R^2 and p-values, see also Table 1).

Table S1

							Cell concentration (Cells L-1)								
Latitude (°N)	Longitude (°E)	Date	Tran -sect	Sta- tion	Total depth (m)	Depth (m)	Karenia	C. lineatu m	C. fusus	C. ma- crocero s	C. tripos	C. furca	C. hor- ridum	C. longipe s	Probosci a alata
57° 17.248’	7° 45.347’	2016-07-21	2	62	53	5	320	0	20	0	60	0	0	0	380
57° 17.248’	7° 45.347’	2016-07-21	2	62	53	15	100	0	0	60	60	0	0	0	2940
57° 17.248’	7° 45.347’	2016-07-21	2	62	53	21	200	0	40	20	240	0	0	0	760
57° 17.248’	7° 45.347’	2016-07-21	2	62	53	34	200	0	40	0	140	0	20	80	100
57° 17.248’	7° 45.347’	2016-07-21	2	62	53	35	220	0	20	20	740	0	80	120	190
57° 17.248’	7° 45.347’	2016-07-21	2	62	53	38	60	0	40	0	120	0	40	20	230
57° 6.771’	7° 44.480’	2016-07-15	2	22	41	15	236962	4360	120	60	0	60	0	0	250
57° 28.801’	7° 46.095’	2016-07-17	2	41	167	5	60	0	60	40	60	40	0	0	730
57° 28.801’	7° 46.095’	2016-07-17	2	41	167	26	4340	0	180	0	180	0	60	80	780
57° 43.145’	7° 15.508’	2016-07-19	3	51	360	5	40	0	20	40	120	20	0	0	300
57° 43.145’	7° 15.508’	2016-07-19	3	51	360	22	17320	0	200	0	280	100	220	200	2140
56° 20.630’	6° 17.569’	2016-07-26	5	103	46	5	80	40	0	0	0	0	0	0	10
56° 20.630’	6° 17.569’	2016-07-26	5	103	46	24	155866	14360	60	0	0	20	0	0	100
57° 28.774’	7° 45.698’	2016-07-14	2	13	151	5	120	0	20	20	60	0	0	0	530
57° 28.774’	7° 45.698’	2016-07-14	2	13	151	35	0	0	0	0	200	0	20	20	90
57° 28.774’	7° 45.698’	2016-07-14	2	13	151	50	0	0	0	0	0	0	0	0	30
57° 12.969’	7° 45.971’	2016-07-15	2	21	53	5	740	0	20	0	0	20	0	0	230
57° 12.969’	7° 45.971’	2016-07-15	2	21	53	16	143705	3740	100	0	20	40	0	0	50
57° 12.969’	7° 45.971’	2016-07-15	2	21	53	35	92336	1240	0	0	0	40	0	0	50

57° 8.950’	7° 45.579’	2016-07-15	2	23	45	15	89080	840	40	20	0	40	0	0	150
57° 10.922’	7° 45.949’	2016-07-16	2	25	52	19	230814	120	60	40	20	20	0	0	0
57° 12.348’	7° 46.849’	2016-07-16	2	26	53	19	238328	120	60	160	40	0	0	0	150
57° 14.415’	7° 46.225’	2016-07-16	2	27	53	16	97257	0	20	40	160	40	0	0	40
57° 16.148’	7° 45.429’	2016-07-16	2	28	54	28	6140	0	1700	20	1580	20	160	320	0
57° 16.301’	7° 45.214’	2016-07-16	2	29	54	18	26320	0	20	20	80	0	0	20	360
57° 16.976’	7° 45.463’	2016-07-16	2	30	57	17	1520	0	40	0	120	0	20	0	870
57° 16.976’	7° 45.463’	2016-07-16	2	30	57	24	500	0	1540	0	1140	0	80	200	40
57° 18.229’	7° 46.419’	2016-07-16	2	31	59	15	200	0	20	20	80	0	0	0	960
57° 18.229’	7° 46.419’	2016-07-16	2	31	59	22	260	0	40	0	360	0	60	40	70
57° 18.061’	7° 46.521’	2016-07-16	2	32	63	18	320	20	20	0	180	0	0	0	1580
57° 18.061’	7° 46.521’	2016-07-16	2	32	63	25	400	0	120	20	360	0	40	40	90
57° 20.920’	7° 46.155’	2016-07-16	2	34	71	15	200	0	60	20	120	20	0	0	720
57° 20.920’	7° 46.155’	2016-07-16	2	34	71	32	140	0	0	0	20	0	40	20	90
57° 21.770’	7° 46.442’	2016-07-17	2	36	74	5	140	0	20	20	20	0	20	0	960
57° 21.770’	7° 46.442’	2016-07-17	2	36	74	36	220	0	100	0	40	0	40	80	110
57° 23.477’	7° 45.861’	2016-07-17	2	38	105	16	200	60	40	200	120	0	0	0	1760
57° 23.477’	7° 45.861’	2016-07-17	2	38	105	37	0	0	20	0	120	0	80	60	40
57° 25.245’	7° 46.232’	2016-07-17	2	39	120	30	560	0	80	0	140	0	120	60	50
57° 27.468’	7° 47.126’	2016-07-17	2	40	146	26	620	0	320	0	80	0	0	120	80
57° 0.027’	7° 15.994’	2016-07-18	3	43	36	5	20	0	20	0	120	0	0	0	690
57° 0.027’	7° 15.994’	2016-07-18	3	43	36	23	130319	174570	120	0	40	660	40	20	90
57° 45.951’	6° 45.994’	2016-07-	4	127	340	5	1780	0	0	20	60	20	0	0	300

		28													
57° 45.951’	6° 45.994’	2016-07-28	4	127	340	10	209338	140	40	40	80	0	0	0	60
57° 45.951’	6° 45.994’	2016-07-28	4	127	340	13	222990	160	80	0	60	40	0	0	190
57° 45.951’	6° 45.994’	2016-07-28	4	127	340	33	32440	500	80	0	40	20	40	120	3310
57° 43.280’	6° 46.563’	2016-07-28	4	126	322	5	6220	20	40	20	100	0	0	0	210
57° 43.280’	6° 46.563’	2016-07-28	4	126	322	28	12160	60	440	0	180	60	160	280	19680
57° 43.280’	6° 46.563’	2016-07-28	4	126	322	40	0	0	0	0	0	0	0	0	240
57° 40.491’	6° 46.792’	2016-07-28	4	125	301	5	0	0	0	20	40	0	0	0	110
57° 40.491’	6° 46.792’	2016-07-28	4	125	301	10	2260	0	20	0	0	0	0	0	410
57° 40.491’	6° 46.792’	2016-07-28	4	125	301	20	8360	0	440	20	80	40	0	0	2440
57° 40.491’	6° 46.792’	2016-07-28	4	125	301	25	3980	0	360	0	600	0	160	140	29280
57° 37.765’	6° 46.683’	2016-07-24	4	82	319	0	3000	0	200	0	260	20	20	40	4520
57° 37.765’	6° 46.683’	2016-07-24	4	82	319	0	13180	0	500	0	320	100	400	120	32680
57° 38.039’	6° 46.667’	2016-07-28	4	124	320	5	100	0	0	100	80	20	0	0	120
57° 38.039’	6° 46.667’	2016-07-28	4	124	320	10	80	0	20	40	20	0	0	0	540
57° 38.039’	6° 46.667’	2016-07-28	4	124	320	16	93600	0	580	20	100	20	0	20	690
57° 38.039’	6° 46.667’	2016-07-28	4	124	320	20	24360	60	300	0	240	0	20	160	6480
57° 35.246’	6° 46.164’	2016-07-28	4	123	210	5	80	0	0	60	20	60	0	0	130
57° 35.246’	6° 46.164’	2016-07-28	4	123	210	10	180	0	100	40	40	0	0	0	430
57° 35.246’	6° 46.164’	2016-07-28	4	123	210	20	4940	0	420	20	560	40	180	420	10960
57° 35.246’	6° 46.164’	2016-07-28	4	123	210	23	10600	0	400	60	260	40	220	100	11060
57° 27.394’	6° 46.481’	2016-07-27	4	120	126	5	40	0	0	0	60	0	0	0	210
57° 27.394’	6° 46.481’	2016-07-	4	120	126	10	100	0	0	40	0	0	0	0	280

		27													
57° 27.394’	6° 46.481’	2016-07-27	4	120	126	20	540	20	260	0	160	0	0	20	6030
57° 27.394’	6° 46.481’	2016-07-27	4	120	126	35	60	0	80	100	140	0	80	140	410
57° 24.389’	6° 46.271’	2016-07-27	4	119	112	5	80	0	0	0	80	0	20	0	700
57° 24.389’	6° 46.271’	2016-07-27	4	119	112	10	20	0	0	0	60	0	0	0	270
57° 24.389’	6° 46.271’	2016-07-27	4	119	112	20	260	20	80	0	60	0	20	0	2840
57° 24.389’	6° 46.271’	2016-07-27	4	119	112	35	40	0	160	20	720	0	20	160	1040
57° 22.069’	6° 46.705’	2016-07-27	4	118	96	5	0	20	0	20	20	0	0	0	520
57° 22.069’	6° 46.705’	2016-07-27	4	118	96	10	260	0	0	60	20	20	0	0	640
57° 22.069’	6° 46.705’	2016-07-27	4	118	96	35	60	0	120	480	300	0	100	80	1520
57° 22.069’	6° 46.705’	2016-07-27	4	118	96	50	0	0	0	0	20	0	0	60	160
57° 18.714’	6° 46.411’	2016-07-29	4	130	83	5	80	0	40	80	20	0	0	0	650
57° 18.714’	6° 46.411’	2016-07-29	4	130	83	10	80	0	0	0	20	0	0	0	480
57° 18.714’	6° 46.411’	2016-07-29	4	130	83	20	240	0	20	0	0	0	0	0	720
57° 18.714’	6° 46.411’	2016-07-29	4	130	83	38	40	0	60	0	400	0	60	140	80
57° 11.219’	6° 46.364’	2016-07-27	4	114	66	5	40	20	20	0	0	0	0	0	700
57° 11.219’	6° 46.364’	2016-07-27	4	114	66	10	80	0	0	0	40	0	0	0	670
57° 11.219’	6° 46.364’	2016-07-27	4	114	66	20	2900	0	20	40	80	0	0	20	70
57° 11.219’	6° 46.364’	2016-07-27	4	114	66	30	640	160	1020	20	320	0	0	100	90
57° 11.219’	6° 46.364’	2016-07-27	4	114	66	40	120	100	40	0	100	0	0	460	80
57° 5.771’	6° 46.291’	2016-07-27	4	113	59	5	280	60	0	0	0	0	0	0	210
57° 5.771’	6° 46.291’	2016-07-27	4	113	59	10	240	20	40	20	20	0	0	0	250
57° 5.771’	6° 46.291’	2016-07-27	4	113	59	20	217681	40	80	100	0	0	0	0	40

57° 5.771’	6° 46.291’	2016-07-27	4	113	59	30	25240	880	180	20	100	20	0	100	10
57° 5.771’	6° 46.291’	2016-07-27	4	113	59	54	8940	40	20	20	0	0	0	0	180
57° 0.145’	6° 46.473’	2016-07-27	4	112	45	5	100	300	0	20	120	0	20	0	120
57° 0.145’	6° 46.473’	2016-07-27	4	112	45	10	120	20	40	0	140	0	0	0	90
57° 0.145’	6° 46.473’	2016-07-27	4	112	45	21	323108	3660	120	80	120	0	0	0	170
57° 0.145’	6° 46.473’	2016-07-27	4	112	45	40	28360	1780	0	0	0	40	0	0	0
56° 49.387’	6° 45.935’	2016-07-23	4	72	37	5	0	0	0	0	160	0	0	0	490
56° 49.387’	6° 45.935’	2016-07-23	4	72	37	17	11440	9300	40	40	60	20	0	0	210
56° 49.387’	6° 45.935’	2016-07-23	4	72	37	25	529749	6580	40	40	20	0	0	0	420
56° 49.387’	6° 45.935’	2016-07-23	4	72	37	27	418951	170306	200	60	60	420	0	20	90
56° 49.387’	6° 45.935’	2016-07-23	4	72	37	32	76240	76080	60	0	0	80	0	0	140
56° 49.232’	6° 45.888’	2016-07-27	4	110	38	10	80	0	0	60	40	20	0	0	440
56° 49.232’	6° 45.888’	2016-07-27	4	110	38	20	280729	7640	60	40	20	20	40	0	130
56° 49.232’	6° 45.888’	2016-07-27	4	110	38	23	741531	257969	1160	60	140	420	200	20	100
56° 49.232’	6° 45.888’	2016-07-27	4	110	38	32	90037	88849	60	0	0	180	0	0	100
56° 27.613’	6° 46.227’	2016-07-26	4	106	44	5	840	180	20	0	40	0	0	0	110
56° 27.613’	6° 46.227’	2016-07-26	4	106	44	28	9940	330693	1120	0	0	780	100	0	210
56° 22.358’	6° 47.058’	2016-07-26	4	105	40	5	20	60	0	0	40	0	0	0	40
56° 22.358’	6° 47.058’	2016-07-26	4	105	40	27	36480	3920	20	0	0	0	20	0	220
56° 54.818’	6° 46.331’	2016-07-27	4	111	43	5	80	80	40	60	60	0	0	0	300
56° 54.818’	6° 46.331’	2016-07-27	4	111	43	10	20	500	0	0	40	0	0	0	390
56° 54.818’	6° 46.331’	2016-07-27	4	111	43	20	1800	680	0	0	40	0	0	0	250
56° 54.818’	6° 46.331’	2016-07-27	4	111	43	27	102393	92566	0	60	20	120	40	0	130

56° 54.818’	6° 46.331’	2016-07-27	4	111	43	38	68520	41000	180	0	40	80	60	0	190
56° 16.755’	6° 46.409’	2016-07-27	4	116	73	5	0	20	0	0	40	0	0	0	580
56° 16.755’	6° 46.409’	2016-07-27	4	116	73	10	20	0	0	0	20	0	0	0	530
56° 16.755’	6° 46.409’	2016-07-27	4	116	73	20	400	20	20	20	80	0	0	0	2160
56° 16.755’	6° 46.409’	2016-07-27	4	116	73	37	0	20	20	0	120	0	0	40	12800
56° 16.755’	6° 46.409’	2016-07-27	4	116	73	45	60	0	0	0	0	0	0	0	220
57° 48.834’	6° 45.673’	2016-07-19	4	52	358	16	36360	20	180	200	200	0	0	20	460
57° 48.834’	6° 45.673’	2016-07-19	4	52	358	33	500	0	200	0	20	40	0	140	7400
57° 32.652’	6° 45.934’	2016-07-19	4	55	175	16	36280	0	80	200	40	40	20	0	1010
57° 32.652’	6° 45.934’	2016-07-19	4	55	175	31	6440	0	320	0	840	80	140	100	2740
57° 26.935’	6° 46.233’	2016-07-20	4	56	122	19	18880	0	40	80	160	0	0	0	1430
57° 21.716’	6° 46.448’	2016-07-20	4	57	96	22	560	0	80	100	260	0	40	40	1420
57° 16.539’	6° 46.869’	2016-07-20	4	58	70	35	100	0	100	20	480	0	80	120	130
57° 10.921’	6° 45.958’	2016-07-20	4	59	66	37	2080	0	160	0	140	0	20	0	130
57° 48.812’	6° 44.718’	2016-07-24	4	84	356	5	60	0	0	20	20	60	0	0	180
57° 48.812’	6° 44.718’	2016-07-24	4	84	356	34	34520	20	580	0	300	140	220	60	13430
56° 43.822’	6° 46.187’	2016-07-27	4	109	41	5	120	20	0	20	80	20	0	0	100
56° 43.822’	6° 46.187’	2016-07-27	4	109	41	24	307180	162425	460	40	20	480	120	20	70
57° 18.787’	6° 46.015’	2016-07-30	4	132	82	5	40	0	0	20	40	0	0	0	560
57° 18.787’	6° 46.015’	2016-07-30	4	132	82	10	60	0	0	20	0	0	0	0	380
57° 18.787’	6° 46.015’	2016-07-30	4	132	82	20	340	0	40	20	20	0	20	0	1330
57° 18.787’	6° 46.015’	2016-07-30	4	132	82	32	140	0	100	40	1160	0	40	220	370

57° 18.787’	6° 46.015’	2016-07-30	4	132	82	40	0	0	0	0	20	0	0	0	640
57° 18.787’	6° 46.015’	2016-07-30	4	132	82	44	0	0	0	0	40	0	0	0	490
57° 52.549’	6° 16.149’	2016-07-24	5	85	307	5	80	0	0	0	40	0	0	0	30
57° 52.549’	6° 16.149’	2016-07-24	5	85	307	19.5	112892	40	200	20	180	60	0	40	890
57° 52.549’	6° 16.149’	2016-07-24	5	85	307	26.1	109453	20	560	0	520	60	160	260	1910
57° 52.549’	6° 16.149’	2016-07-24	5	85	307	31.3	38640	0	360	20	260	100	240	100	3160

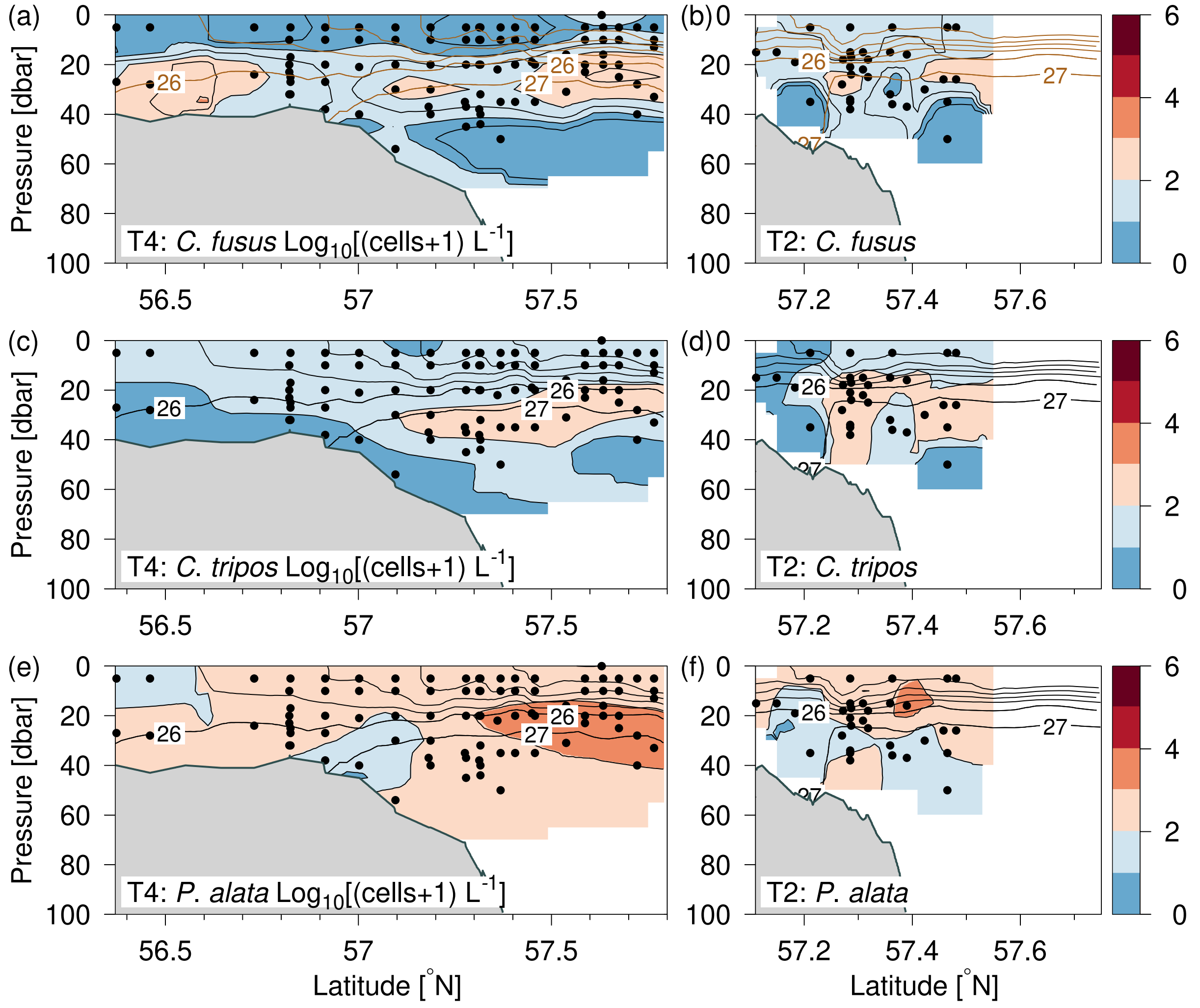

Figure S1 Distributions along transect 4 (T4: 6.75 °E) and 2 (T2: 7.75 °E): (a, b) concentration of *Ceratium fusus* and density contours (brown lines, σ_θ, intervals of 1 kg m^{-3}), (c, d) concentration of *Ceratium tripos* and (e, f) *Proboscia alata*.

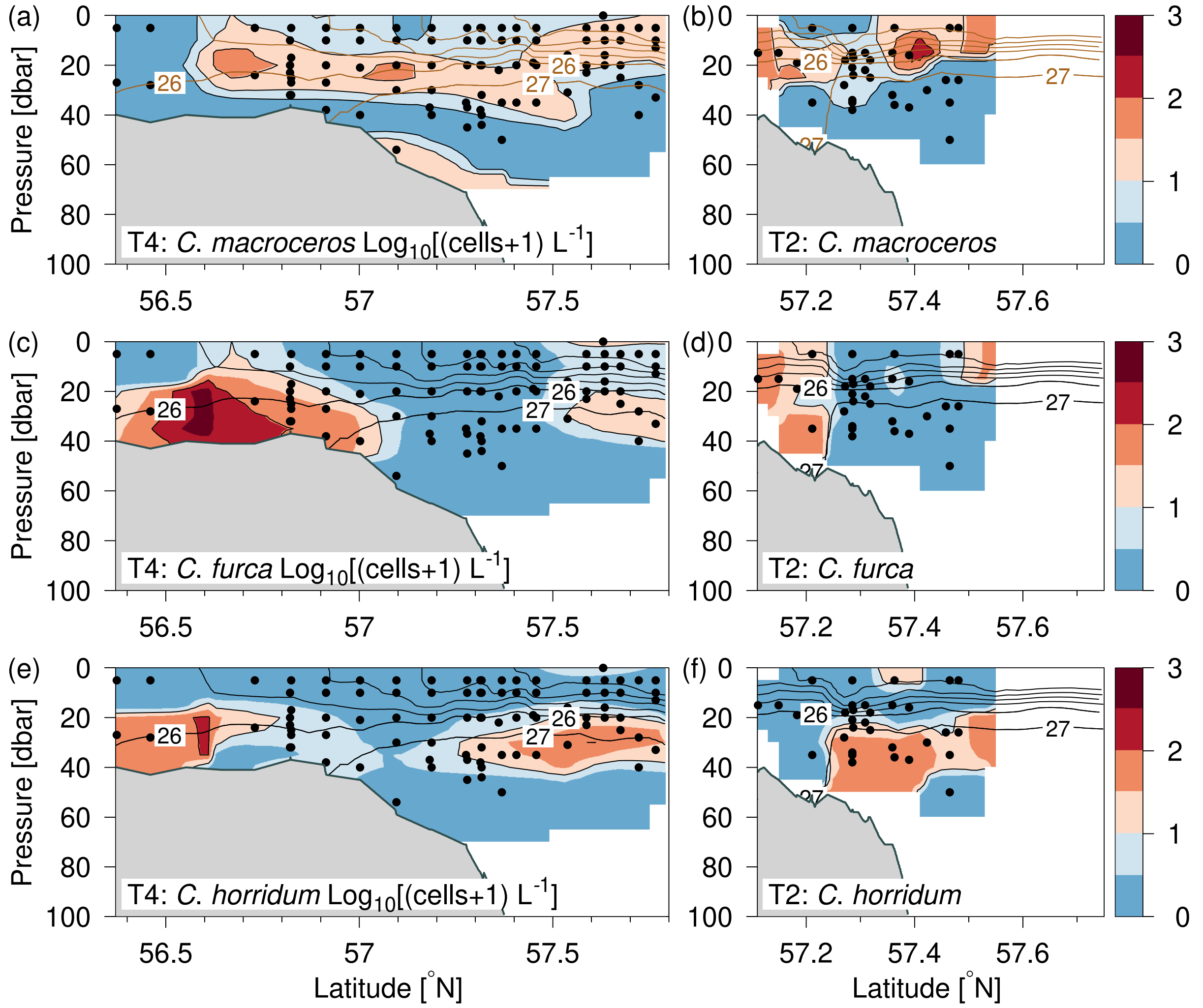

Figure S2 Distributions along transect 4 (T4: 6.75 °E) and 2 (T2: 7.75 °E): (a, b) concentration of *Ceratium macroceros* and density contours (brown lines, σ_θ, intervals of 1 kg m^{-3}), (c, d) concentration of *Ceratium furca* and (e, f) *Ceratium horridum*.

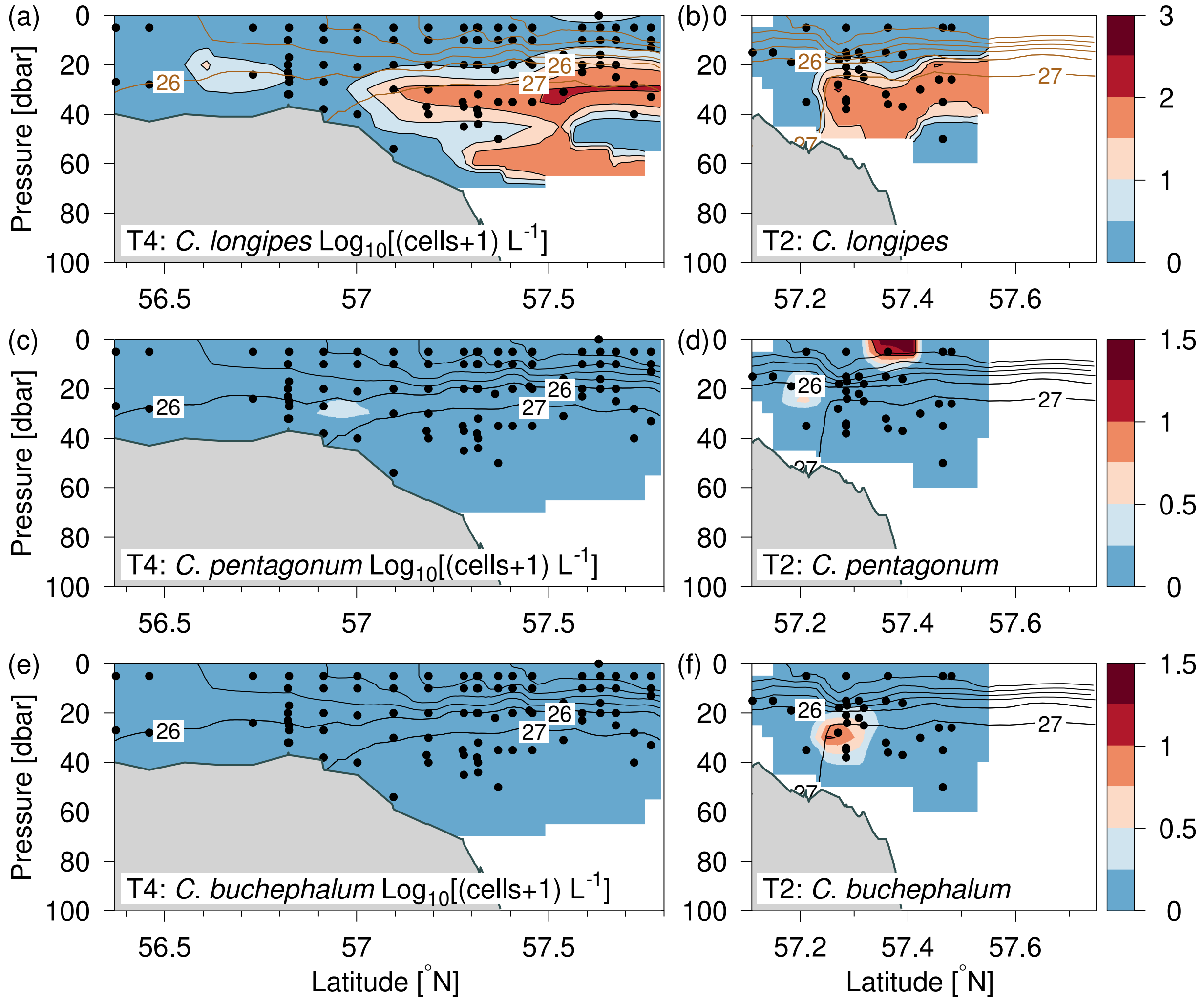

Figure S3 Distributions along transect 4 (T4: 6.75 °E) and 2 (T2: 7.75 °E): (a, b) concentration of *Ceratium longipes* and density contours (brown lines, σ_θ, intervals of 1 kg m^{-3}), (c, d) concentration of *Ceratium pentagonum* and (e, f) *Ceratium buchephalum*.

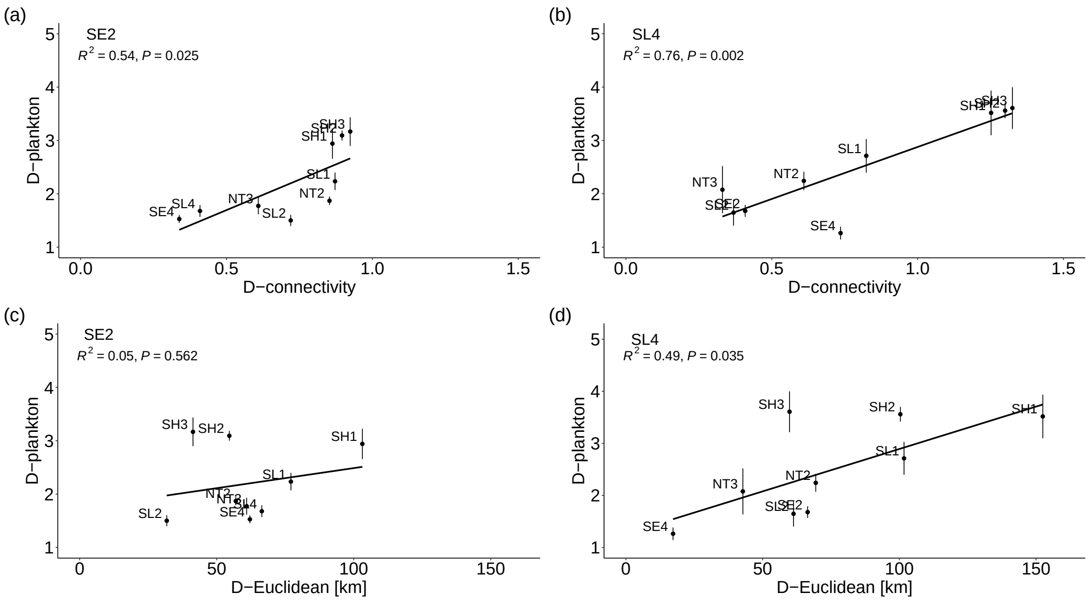

Figure S4 Average dissimilarity distances in ecological space (bullets and standard errors) versus connectivity distance between samples in (a) SE2 (also shown in Figure 4c), (b) SL4, and (c, d) the corresponding relation to Euclidean distances (linear regression, R2 and p-values, see also Table 1).
